# Supplementary material for: Cancer Incidence in Korean Healthcare Workers in Hospitals
Source: Cancers (Basel). 2023 Mar 29;15(7):2045. doi: 10.3390/cancers15072045 (PMC10093411; doi:10.3390/cancers15072045)
Supplement: Supplementary file 1 [file cancers-15-02045-s001.zip › cancers-2255563-supplementary.pdf]

Article

Cancer Incidence in Korean Healthcare Workers in Hospitals

Dong-Wook Lee [1,2], Hyeonjun Kim [3], Wanhyung Lee [4], Woo-Ri Lee [5], Ki-Bong Yoo [6], Jun-Hyeok Choi [7],
Kyung-Eun Lee [7] and Jin-Ha Yoon [8,*]

[1] Department of Occupational and Environmental Medicine, Inha University Hospital, Inha University, Incheon 22332, Republic of Korea
[2] Jungbu Korea Occupational Diseases Surveillance Center, Incheon 22332, Republic of Korea
[3] Department of Occupational and Environmental Medicine, Chonnam National University Medical School and Chonnam National University Hwasun Hospital, Jeollanam-do 58128, Republic of Korea
[4] Department of Occupational and Environmental Medicine, Gil Medical Center, Gachon University College of Medicine, Incheon 21654, Republic of Korea
[5] Department of Research and Analysis, National Health Insurance Service Ilsan Hospital, Gyeonggi-do 10444, Republic of Korea
[6] Division of Health Administration, College of Software and Digital Healthcare Convergence, Yonsei University, Gangwon-do 26426, Republic of Korea
[7] Occupational Safety and Health Research Institute, Korea Occupational Safety and Health Agency, Ulsan 44429, Republic of Korea
[8] Department of Occupational and Environmental Medicine, Severance Hospital, Yonsei University College of Medicine, Seoul 03722, Republic of Korea
* Correspondence: flyinyou@yuhs.ac

Table of Contents

Supplementary Table S1. Descriptive statistics of the cohort…………….……………… 3

Supplementary Table S2. Observed Number of Deaths, Standardized Incidence Ratio and 95% Confidence Interval, Total Workers as the Reference Group (Dynamic cohort)…..4

Supplementary Table S3. Observed Number of Deaths, Standardized Incidence Ratio and 95% Confidence Interval, Public Officials as the Reference Group (Dynamic cohort)…..5

Supplementary Table S4. The summary of significant findings in this study……………6

Supplementary Table S1. Descriptive statistics of the cohort

Variable	HHCWs		Total workers		Public officials	
	Fixed cohort	Dynamic cohort	Fixed cohort	Dynamic cohort	Fixed cohort	Dynamic cohort
	n (%) mean ± SD	n (%) mean ± SD	n (%) mean ± SD	n (%) mean ± SD	n (%) mean ± SD	n (%) mean ± SD
Total	107,646	363,847	8,317,710	11,050,398	611,833	677,667
Person-years						
Total	905,303	2,463,244	68,787,462	75,474,218	4,288,949	4,723,339
Average	8.41 (±0.84)	6.77 (±0.97)	8.27 (±0.98)	6.83 (±0.84)	7.01 (±0.28)	6.97 (±0.33)
Gender						
Male	37,243	195,846	5,740,042	7,362,615	300,080	338,793
Female	70,403	168,001	2,577,668	3,687,783	311,753	338,874
Age at end of follow-up (yrs)						
25-34	52,469	118,604	2,866,046	3,760,839	167,944	182,043
35-44	30,738	117,893	2,824,660	3,622,940	208,193	222,986
45-54	17,316	89,027	1,993,575	2,641,425	187,779	203,699
55-64	7,123	38,323	633,429	1,025,194	47,917	68,939
Income						
1Q (lowest)	21,064	188,949	1,871,217	2,816,275	17,789	19,886
2Q	37,648	83,645	1,924,848	2,705,880	45,171	49,849
3Q	27,830	57,491	2,204,875	2,758,013	194,982	210,576
4Q(highest)	21,104	33,762	2,316,770	2,770,230	353,891	397,356

HHCWs, healthcare workers in hospitals; SD, standard deviation; Q1–4, Quartile 1–4

Supplementary Table S2. Observed Number of Deaths, Standardized Incidence Ratio and 95% Confidence Interval, Total Workers as the Reference Group (Dynamic cohort)

	Male					Female				
Cancer classification	**Obs**	**Exp**	**SIR**	**95% CI**	***P***	**Obs**	**Exp**	**SIR**	**95% CI**	***P***
All-cause death	252	297.9	0.85	(0.76-0.95)	0.004	281	247.5	1.14	(1.00-1.29)	0.020
[C00-C97] Malignant neoplasm	545	600.9	0.91	(0.84-0.98)	0.011	2094	1973.2	1.06	(1.02-1.11)	0.004
[C00-C14] Lip, oral cavity and pharynx	17	12.7	1.34	(0.78-2.54)	0.144	19	11.6	1.64	(0.93-3.22)	0.028
[C15-C26] Digestive organs	288	311.0	0.93	(0.83-1.04)	0.100	299	286.3	1.04	(0.93-1.18)	0.234
(C16) Stomach	109	117.1	0.93	(0.78-1.13)	0.244	132	119.2	1.11	(0.93-1.34)	0.131
(C18) Colon	51	46.6	1.09	(0.82-1.49)	0.278	56	61.2	0.91	(0.71-1.20)	0.279
(C22) Liver and intrahepatic bile ducts	51	70.5	0.72	(0.57-0.93)	0.009	30	27.4	1.10	(0.76-1.66)	0.334
(C25) Pancreas	9	14.0	0.64	(0.38-1.17)	0.109	17	14.7	1.16	(0.70-2.09)	0.307
[C30-C39] Respiratory and intrathoracic organs	60	58.6	1.02	(0.79-1.35)	0.445	76	61.0	1.25	(0.97-1.63)	0.035
(C33-C34) Trachea & Malignant neoplasm of bronchus and lung	51	50.0	1.02	(0.77-1.38)	0.462	70	54.4	1.29	(0.99-1.71)	0.024
[C40-C41] Bone and articular cartilage	4	3.0	1.34	(0.46-6.53)	0.353	7	5.6	1.25	(0.56-3.56)	0.330
[C43-C44] Skin	3	7.6	0.39	(0.20-0.94)	0.055	16	12.5	1.28	(0.74-2.44)	0.194
[C45-C49] Mesothelial and soft tissue	5	6.6	0.76	(0.36-1.97)	0.355	28	15.2	1.84	(1.12-3.27)	0.002
[C50-C50] Breast	0					501	442.3	1.13	(1.03-1.25)	0.003
[C51-C58] Female genital organs						191	175.4	1.09	(0.94-1.27)	0.128
[C60-C63] Male genital organs	24	32.1	0.75	(0.53-1.09)	0.085					
[C64-C68] Urinary tract	40	45.3	0.88	(0.66-1.21)	0.242	30	25.6	1.17	(0.80-1.80)	0.216
[C70-C72] Brain and other parts of central nervous system	8	9.5	0.84	(0.45-1.80)	0.392	16	17.7	0.90	(0.57-1.53)	0.402
[C81-C96] Lymphoid, haematopoietic and related tissue	42	35.7	1.18	(0.85-1.68)	0.165	66	56.1	1.18	(0.91-1.56)	0.107
(C82-C85) Non-Hodgkin's lymphoma	21	16.2	1.3	(0.80-2.26)	0.143	28	24.4	1.15	(0.77-1.78)	0.258
(C91-C95) Leukaemia	11	11.8	0.93	(0.53-1.82)	0.485	27	20.0	1.35	(0.87-2.21)	0.078

Obs, observed cases; Exp, expected cases; SIR: standardized incidence ratio; CI: confidence interval.
Those with $P < 0.002$ (significant under Bonferroni correction) were in bold.

Supplementary Table S3. Observed Number of Deaths, Standardized Incidence Ratio and 95% Confidence Interval, Public Officials as the Reference Group (Dynamic cohort)

	Male					Female				
Cancer classification	**Obs**	**Exp**	**SIR**	**95% CI**	***P***	**Obs**	**Exp**	**SIR**	**95% CI**	***P***
All-cause death	**252**	**188.9**	**1.33**	**(1.16-1.55)**	**<0.001**	**281**	**212.3**	**1.32**	**(1.16-1.52)**	**<0.001**
[C00-C97] Malignant neoplasm	545	609.4	0.89	(0.83-0.97)	0.004	2094	2216.1	0.94	(0.91-0.99)	0.005
[C00-C14] Lip, oral cavity and pharynx	17	12.7	1.33	(0.78-2.53)	0.144	19	13.0	1.46	(0.85-2.74)	0.070
[C15-C26] Digestive organs	288	295.2	0.98	(0.87-1.10)	0.351	299	284.8	1.05	(0.93-1.18)	0.207
(C16) Stomach	109	114.2	0.95	(0.79-1.16)	0.335	132	123.9	1.07	(0.89-1.28)	0.245
(C18) Colon	51	47.6	1.07	(0.81-1.45)	0.330	56	60.9	0.92	(0.72-1.20)	0.291
(C22) Liver and intrahepatic bile ducts	51	61.3	0.83	(0.65-1.09)	0.103	30	25.4	1.18	(0.80-1.82)	0.205
(C25) Pancreas	9	13.6	0.66	(0.39-1.23)	0.130	17	16.8	1.01	(0.63-1.74)	0.513
[C30-C39] Respiratory and intrathoracic organs	60	49.0	1.22	(0.93-1.65)	0.070	76	68.8	1.11	(0.87-1.42)	0.208
(C33-C34) Trachea & Malignant neoplasm of bronchus and lung	51	41.7	1.22	(0.90-1.70)	0.090	70	61.6	1.14	(0.89-1.48)	0.157
[C40-C41] Bone and articular cartilage	4	2.6	1.54	(0.49-8.78)	0.264	7	6.2	1.13	(0.52-3.01)	0.426
[C43-C44] Skin	3	7.4	0.41	(0.20-0.98)	0.063	16	12.8	1.25	(0.73-2.36)	0.219
[C45-C49] Mesothelial and soft tissue	5	7.2	0.7	(0.34-1.72)	0.276	28	17.4	1.61	(1.01-2.75)	0.012
[C50-C50] Breast	0					501	533.0	0.94	(0.86-1.03)	0.085
[C51-C58] Female genital organs						191	156.1	1.22	(1.05-1.44)	0.004
[C60-C63] Male genital organs	**24**	**42.3**	**0.57**	**(0.42-0.79)**	**0.0016**					
[C64-C68] Urinary tract	40	45.6	0.88	(0.66-1.20)	0.228	30	26.6	1.13	(0.77-1.72)	0.279
[C70-C72] Brain and other parts of central nervous system	8	8.4	0.95	(0.49-2.15)	0.537	16	21.6	0.74	(0.49-1.19)	0.134
[C81-C96] Lymphoid, haematopoietic and related tissue	42	35.3	1.19	(0.86-1.71)	0.149	66	53.7	1.23	(0.94-1.64)	0.057
(C82-C85) Non-Hodgkin's lymphoma	21	15.1	1.39	(0.84-2.48)	0.087	28	22.4	1.25	(0.83-1.98)	0.141
(C91-C95) Leukaemia	11	12.0	0.92	(0.53-1.78)	0.462	27	17.6	1.53	(0.97-2.60)	0.022

Obs, observed cases; Exp, expected cases; SIR: standardized incidence ratio; CI: confidence interval.
Those with $P < 0.002$ (significant under Bonferroni correction) were in bold.

Supplementary Table S4. The summary of significant findings in this study

Classification	Male HHCWs			Female HHCWs		
	Control group	SIR	95% CI	Control group	SIR	95% CI
All-cause death						
	Public officials	1.50	(1.25-1.80)	Total worker	1.25	(1.06-1.47)
				Public officials	1.62	(1.36-1.96)
[C00-C97] Malignant neoplasm						
	Public officials	1.15	(1.05-1.28)	Total worker	1.09	(1.03-1.15)
				Public officials	1.15	(1.09-1.22)
[C15-C26] Digestive organs						
	Public officials	1.30	(1.13-1.51)	Public officials	1.34	(1.15-1.58)
[C30-C39] Respiratory and intrathoracic organs						
	Public officials	1.84	(1.29-2.72)			
(C33-C34) Trachea & Malignant neoplasm of bronchus and lung						
	Public officials	1.84	(1.25-2.81)			
[C45-C49] Mesothelial and soft tissue						
				Public officials	2.19	(1.16-4.75)
[C50-C50] Breast						
				Total worker	1.21	(1.09-1.36)
				Public officials	1.18	(1.06-1.32)
[C51-C58] Female genital organs						
				Public officials	1.49	(1.21-1.85)
[C60-C63] Male genital organs						
	Public officials	0.57	(0.42-0.79)			
(C82-C85) Non-Hodgkin's lymphoma						
	Public officials	2.19	(1.14-4.89)			

HHCW, healthcare workers in hospital; SIR, standardized incidence ratio; CI, confidence interval
The "Fixed" job definition of HHCWs and control groups were based on participants' workplace for at least three consecutive years from 2007 to 2015
Those with $P < 0.002$ were regarded as significant under Bonferroni correction.
